# Genetic Determinants of Financial Risk Taking

**DOI:** 10.1371/journal.pone.0004362

**Published:** 2009-02-11

**Authors:** Camelia M. Kuhnen, Joan Y. Chiao

**Affiliations:** 1 Kellogg School of Management, Northwestern University, Evanston, Illinois, United States of America; 2 Department of Psychology, Northwestern University, Evanston, Illinois, United States of America; 3 Northwestern University Interdepartmental Neuroscience Program, Evanston, Illinois, United States of America; University of Utah, United States of America

## Abstract

Individuals vary in their willingness to take financial risks. Here we show that variants of two genes that regulate dopamine and serotonin neurotransmission and have been previously linked to emotional behavior, anxiety and addiction (5-HTTLPR and DRD4) are significant determinants of risk taking in investment decisions. We find that the 5-HTTLPR *s/s* allele carriers take 28% less risk than those carrying the *s/l* or *l/l* alleles of the gene. DRD4 7-repeat allele carriers take 25% more risk than individuals without the 7-repeat allele. These findings contribute to the emerging literature on the genetic determinants of economic behavior.

## Introduction

Risk preferences describe individuals' willingness to take or avoid risk in a variety of settings, including financial choice, and are an essential component of any model of economic behavior. Individuals vary in the extent to which they are willing to take financial risks, which may be explained in part by individual differences in heritable traits. Classical twin design studies estimate that genetic effects account for 20% variation in risk taking in experimental lottery choices [1] and between 35–54% of the liability for developing symptoms of pathological gambling [2]. However, identification of specific genes underlying financial risk preferences has remained elusive.

Recent findings in neuroscience suggest that the neurotransmitters dopamine [3] and serotonin [4] have important roles in decision making. Genes that regulate these neurotransmitters impact the processing of information about rewarding [5]–[6] and harmful stimuli [6]–[8], are related to personality traits such as extraversion [9], novelty seeking [10] and anxiety [11], and are associated with developing addictions [12]. Moreover, activity within the anterior insula and the nucleus accumbens, brain regions innervated by serotonergic and dopaminergic neural pathways, has been shown to relate to individuals' financial risk taking behavior [13].

Given the mounting evidence that the dopaminergic and serotonergic systems are involved in decision making, and that genetic variations have a significant effect on the physiology of these two systems [14]–[15], we sought to understand whether such genetic variations lead to individual differences in financial risk taking preferences. Prior research suggests that variants of two specific genes may be involved in risk and reward processing, and therefore could influence financial risk taking: the serotonin transporter polymorphism (*5-HTTLPR*) and dopamine D4 receptor (*DRD4*) exon III polymorphism. The *5-HTTLPR* consists of a 44-base pair insertion or deletion, generating either a long *(l)* or a short *(s)* allele. The short variant of the polymorphism reduces the transcriptional efficiency of the *5-HTT* gene promoter and is associated with higher scores on neuroticism and harm avoidance [11]. The dopamine D4 receptor (*DRD4*) exon III polymorphism has been linked to novelty seeking and pathological gambling. Individuals with the 7-repeat allele have higher novelty seeking scores than those with other *DRD4* variants [10] and are more likely to be pathological gamblers [16].

Here we investigated whether or not genetic variations in these two candidate functional polymorphisms, *5-HTTLPR* and *DRD4*, contribute to individual differences in financial risk taking preferences. Based on prior work, we hypothesized that individuals carrying two copies of the s allele of the *5-HTTLPR* would be significantly more risk averse relative to individuals carrying one or two copies of the *l* allele. Additionally, we hypothesized that individuals with a 7-repeat allele of the *DRD4* polymorphism would be significantly more risk seeking relative to those individuals without the 7-repeat allele.

**Figure 1:**
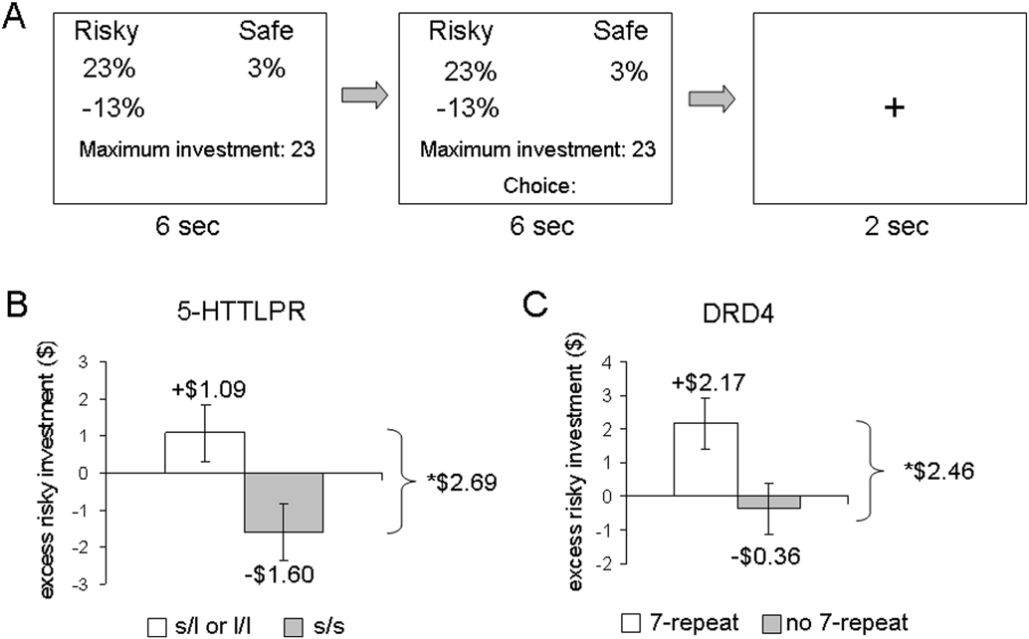
(A) Trial structure of the investment task. For 6 seconds subjects observe the two possible and equally-likely values of the return of the risky asset, the return of the safe asset and the amount they have to invest that trial. When the word “Choice” appears on the screen, subjects have 6 seconds to enter the amount they wish to invest in the risky asset. Their remaining funds are automatically invested in the safe asset. A 2-second fixation screen precedes a new trial. (B) 5-HTTLPR and risk taking propensity. Individuals carrying one or two copies of the l allele demonstrated significantly greater risk taking relative to individuals carrying two copies of the s allele (*p*<0.02). Error bars indicate standard errors. (C) DRD4 and risk taking propensity. Individuals carrying the 7-repeat allele demonstrated significantly greater risk taking relative to individuals without the 7-repeat allele (*p*<0.04). Error bars indicate standard errors.

We elicited financial risk preferences in an experimental setting where participants made multiple investment decisions allocating funds between a risky and a riskless asset, and were compensated based on the performance of their chosen financial portfolio (Fig. 1A). We subsequently genotyped participants for *5-HTTLPR* and *DRD4* functional polymorphisms (see Supplement).

We found that individuals carrying two copies of the short allele of the *5-HTTLPR* are significantly more risk averse relative to individuals carrying one or two copies of the long allele. Additionally, individuals with the 7-repeat allele of *DRD4* are significantly more risk seeking relative to those individuals without the 7-repeat allele. These findings provide novel evidence of a genetic basis for financial choices.

## Methods

65 subjects (26 male; M age = 22.4 yrs; SD age: 4.9 yrs) completed the investment task and were subsequently genotyped for the *5-HTTLPR* and *DRD4* functional polymorphisms. Genotyping was conducted by ACGT Inc. (Wheeling, IL) (see Supplementary Methods S1).

All participants were affiliated with Northwestern University and were recruited via email announcements sent to the subject pool maintained by the Kellogg School of Management at Northwestern University. The experiment was programmed (and data were collected) using the software package E-Prime. Five to nine subjects were in the laboratory solving the investment task at the same time, with each individual in a cubicle separated from the rest of the participants. Participants gave informed consent prior to participating and the study was approved by the IRB committee at Northwestern University. The entire experiment took 1.5 hours to complete and the average pay per subject was $25.

The entire sample consisted of 21 carriers (8 male) homozygous for the *s* allele, 44 carriers (18 male) with one or two copies of the *l* allele of the *5-HTTLPR* polymorphism (see Supplementary Table S1), 15 carriers (8 male) of the 7-repeat allele, and 50 non-carriers (18 male) of 7-repeat allele variant of *DRD4* (see Supplementary Table S2). We conducted statistical analyses to compare expected versus observed allele frequencies for our *5-HTTLPR* sample (see Supplementary Table S1) and found that *5-HTTLPR* genotypes in our sample were distributed according to Hardy-Weinberg equilibrium (Pearson chi-square = 0.39, df = 1, *p*>0.05). Additionally, we conducted statistical analyses for HW equilibrium of *DRD4* genotypes (see Supplementary Table S2) using the Markov Chain algorithm [17] and found that *DRD4* allele frequencies in the current sample were distributed according to HWE (Markov chain algorithm; *p* = 0.36).

Participants first completed the investment task and were then genotyped. On each of the 96 trials (see Fig. 1A) of the task subjects were given an amount of money $T. Subjects could invest $T+$15 (the show-up fee) in two assets, a riskless and a risky one. The amount not invested in the risky asset was automatically invested in the riskless asset (shorting and borrowing were not allowed). The trial endowment amount T was either $8 or $13, and hence the amount subjects could invest (T+$15 show-up fee) was either $23 or $28 per trial. In one version of the task, subjects were informed that the risky asset would pay either of two possible returns with equal probability, and these two possible outcomes for the return were known by the subject in each trial. In another version of the task, subjects were provided with the expected return and standard deviation of the risky asset. These two ways of presenting information about the payoffs of the risky investment are equivalent if subjects have mean-variance preferences (i.e. they like higher expected returns and lower variance), a common assumption in the finance literature which is also supported by our data. The riskless asset paid a known rate of return. Subjects' choices did not differ across these versions of the task (completed by 26 and 39 subjects, respectively), and therefore we combine the data from the two versions.

At the time of making a choice, subjects knew the actual rate of return of the risk-free asset and the two possible outcomes of the risky security, or, equivalently, the expected value and standard deviation of the risky return. These values differed from trial to trial, as did the amount of money available to the subject to invest. The actual rate of return for the risky asset on any trial was not revealed until the end of the experiment. At the end of the experiment, each subject selected a random number between 1 and 96 by picking a ball from an urn. That number determined the trial for which the subject would receive payment. If on any trial a subject chose to invest in the risky asset an amount larger that the maximum investment allowed ($T+$15), or if they did not respond, that trial was marked as invalid. If an invalid trial was selected from the urn, the final payment was only the show-up fee of $15. Subjects therefore had incentives to always enter their choice for the risky investment, and to treat each of the 96 trials as the one that would determine their pay. By deferring information about earnings until the end of the experiment we eliminate wealth effects that may change subjects' choices depending on past outcomes.

In each trial subjects had six seconds to learn the information about the return distribution of the risky security and the return of the safe asset. They had six additional seconds to enter the dollar amount they wished to invest in the risky asset, which was an integer that could range from zero to the maximum investment of $T+$15. A 2-second fixation screen indicated a new trial was about to begin.

## Results

The amount of money participants invested in the risky security (M risky allocation: $9.78; SD risky allocation: $7.16) on each trial depended on the characteristics of the two investment choices, as would be predicted by standard models of economic choice where individuals have mean-variance preferences [18]. Our benchmark model of investment decisions (see Table 1) indicates that all else equal, participants invested significantly more money in the risky asset if its expected return was higher, the standard deviation of its return was lower, or if the return of the safe asset was lower. Moreover, the higher the amount available to participants, the more money they invested in the risky asset. For each portfolio allocation decision of our subjects we calculated the risky investment in excess of the amount predicted by the benchmark model (i.e. the residual term in the regression model in Table 1). For each subject, we obtained the average across all 96 trials of this residual investment. This average is the subject's *excess risky investment* and it measures how risk seeking an individual is relative to the average person in the subject pool.

**Table 1:** Benchmark model of amount invested in risky asset.

Dependent Variable	*Amount invested in risky asset*
	Coefficient/t-stat
*Risky Asset Expected Return*	42.89
	(9.20)***
*Risky Asset Std Dev of Return*	−3.92
	(−2.55)**
*Safe Asset Return*	−70.01
	(−7.81)***
*Available funds*	0.39
	(7.34)***
*Trial Number*	−0.01
	(−1.42)
*Constant*	−2.66
	(−1.64)
Adj. R^2^	0.13
Observations	5987

The dependent variable is the amount invested in the risky asset in each trial. Independent variables include the characteristics of the two investment options in a given trial, the amount of money available to the subject, as well as a task version indicator variable. Standard errors are robust to heteroscedasticity and correlation among error terms in observations belonging to the same subject. T-statistics are in parentheses.

** p<0.05;

*** p<0.01

Results demonstrate that financial risk seeking is correlated with the *5-HTTLPR* and *DRD4* functional polymorphisms. As shown in Fig. 1B, individuals who carry two copies of the short allele of the *5-HTTLPR* polymorphism invest $2.69 (about 28% of the average risky allocation) less in the risky asset than those carrying one or two copies of the long allele of the genotype (*p*<0.02 in a one-tailed mean comparison test), in excess of the benchmark model. Similarly, individuals who carry the 7-repeat allele in the *DRD4* gene invest $2.46 (about 25% of the average risky allocation) more in the risky asset than those lacking the 7-repeat allele (Fig. 1C, *p*<0.04 in a one-tailed mean comparison test).

## Discussion

Our findings show that two functional polymorphisms known to regulate serotonergic and dopaminergic activity in the brain are associated with individual differences in financial risk-seeking behavior. Consistent with our results, a contemporaneous study [19] showed in a male sample a positive correlation between the presence of the 7-repeat allele of the *D4DR* and the propensity to choose risky investment options using a similar experimental setup. The current work complements a growing body of work demonstrating the heritability of economic decision-making [12], [20] and reveals specific genetic determinants of financial choices. While the effects that we document here are suggestive of a causal relationship between individuals' genotype and risk preferences, our data do not allow for causality to be firmly established.

Prior work in behavioral genetics suggests an evolutionary explanation for our findings. For instance, a seminal study [21] found an association between long distance migration and the proportion of long alleles of DRD4, suggesting that DRD4 is important for novelty-seeking behaviors that may have adaptive value [22]. However, the strength association between the DRD4 gene and novelty-seeking behavior may be variable [23]. For instance, a recent behavioral genetics study found that the presence of the 7R allele of the DRD4 is more advantageous among nomadic relative to settled Ariaal men living in northern Kenya [24]. Similarly, individuals carrying one or two copies of the short allele of the 5-HTTLPR have been shown to be more vulnerable to affective disorders (e.g., anxiety and depression) when exposed to stressful experiences [25], suggesting an adaptive benefit of carrying the long allele of the 5-HTTLPR. Here we show that individuals who carry the long alleles of DRD4 and 5-HTTLPR take greater financial risks. We speculate that financial risk taking may result from evolutionarily adaptive mechanisms that encourage novelty-seeking behavior, however future research is needed to determine whether it leads to better economic outcomes and evolutionary benefits, more broadly construed.

## Supporting Information

Methods S1 Supplementary Materials and Methods
(0.03 MB DOC)

Table S1 Allele and genotype frequencies for 5HTTLPR polymorphism
(0.03 MB DOC)

Table S2 Allele and genotype frequencies for DRD4 polymorphism
(0.05 MB DOC)

## References

[1] Cesarini D, Dawes CT, Johannesson M, Lichtenstein P, Wallace B (2009). Genetic variation in preferences for giving and risk-taking.. Quarterly Journal of Economics.

[2] Eisen SA, Lin N, Lyons MJ, Scherrer JF, Griffith K (1998). Familial influences on gambling behavior: an analysis of 3359 twin pairs.. Addiction.

[3] Schultz W (2007). Behavioral dopamine signals.. TRENDS in Neuroscience.

[4] Daw ND, Sham K, Dayan P (2002). Opponent interactions between serotonin and dopamine.. Neural Networks.

[5] Yacubian J, Sommer T, Schroeder K, Glascher J, Kalisch R (2007). Gene-gene interaction associated with neural reward sensitivity.. PNAS.

[6] Frank MJ, Moustafa AA, Haughey HM, Curran T, Hutchison KE (2007). Genetic triple dissociation reveals multiple roles for dopamine in re-inforcement learning.. PNAS.

[7] Kim SJ, Kim YS, Lee HS, Kim SY, Kim CH (2006). An interaction between the serotonin transporter promoter region and dopamine transporter polymorphisms contributes to harm avoidance and reward dependence traits in normal healthy subjects.. Journal of Neural Transmission.

[8] Klein TA, Neumann J, Reuter M, Hennig J, von Cramon DY (2007). Genetically determined differences in learning from errors.. Science.

[9] Reuter M, Hennig J (2005). Association of the functional catechol-o methyltransferase VALI58MET polymorphism with the personality trait of extraversion.. NeuroReport.

[10] Ebstein RP, Novick O, Umansky R, Priel B, Osher Y (1996). Dopamine D4 receptor (D4DR) exon III polymorphism associated with the human personality trait of novelty seeking.. Nature Genetics.

[11] Lesch KP, Bengel D, Heils A, Sabol SZ, Greenberg BD (1996). Association of anxiety-related traits with a polymorphism in the serotonin transporter gene regulatory region.. Science.

[12] Kreek MJ, Nielsen DA, Butelman ER, LaForger S (2005). Genetic influences on impulsivity, risk taking, stress, responsivity and vulnerability to drug abuse and addiction.. Nature Neuroscience.

[13] Kuhnen CM, Knutson B (2005). The neural basis of financial risk taking.. Neuron.

[14] Caldu X, Dreher JC (2007). Hormonal and genetic influences on processing reward and social information.. Annals of the New York Academy of Sciences.

[15] Hariri AR, Mattay VS, Tessitore A, Kolachana B, Fera F (2002). Serotonin transporter genetic variation and the response of the human amygdala.. Science.

[16] Perez de Castro I, Ibez A, Torres P, Siz-Ruiz J, Fernandez-Piqueras J (1997). Genetic association study between pathological gambling and a functional DNA polymorphism at the D4 receptor gene.. Pharmacogenetics.

[17] Guo SW, Thompson EA (1992). Performing the Exact Test of Hardy-Weinberg Proportion for Multiple Alleles.. Biometrics.

[18] Markowitz H (1952). Portfolio selection.. Journal of Finance.

[19] Dreber A, Apicella C, Eisenberg DTA, Garcia JR, Zamore RS (in press). The 7R polymorphism in the dopamine receptor D4 gene (*DRD4*) is associated with financial risk-taking in men.. Evolution and Human Behavior..

[20] Eisenberg DTA, MacKillop J, Modi M, Beauchemin J, Dang D (2007). Examining Impulsivity as an Endophenotype Using a Behavioral Approach: A DRD2 TaqI A and DRD4 48-bp VNTR Association Study.. Behavioral and Brain Functions.

[21] Chen CS, Burton M, Greenberger E, Dmitrieva J (1999). Population migration and the variation of dopamine D4 receptor (DRD4) allele frequencies around the globe.. Evolution and Human Behavior.

[22] Harpending H, Cochran G (2002). In our genes.. Proceedings of the National Academy of Sciences of the United States of America.

[23] Munafo MR, Yalcin B, Willis-Owen SA, Flint J (2008). Association of the dopamine D4 receptor (DRD4) gene and approach-related personality traits: meta-analysis and new data.. Biological Psychiatry,.

[24] Eisenberg DTA, Campbell B, Gray PB, Sorenson MD (2008). Dopamine receptor genetic polymorphisms and body composition in undernourished pastoralists: An exploration of nutrition indices among nomadic and recently settled Ariaal men of northern Kenya.. BMC Evolutionary Biology,.

[25] Caspi A, Sugden K, Moffitt TE, Taylor A, Craig IW (2003). Influence of life stress on depression: moderation by a polymorphism in the 5-HTT gene.. Science,.

