# Supplementary material for: Genetic Determinants of Financial Risk Taking
Source: PLoS One. 2009 Feb 11;4(2):e4362. doi: 10.1371/journal.pone.0004362 (PMC2634960; doi:10.1371/journal.pone.0004362)
Supplement: Methods S1 — Supplementary Materials and Methods (0.03 MB DOC) [file pone.0004362.s001.doc]

**Supplementary Materials and Methods**

***Genotyping Procedure***

Genomic DNA purification. Saliva was collected in the Oragene DNA collection kit (DNA Genotek). The DNA was extracted using the manufacturer's protocol. Briefly, samples were incubated at 50°C for 1 hr to inactivate enzymes. To each sample, Oragene DNA purifier (1/25 original volume) was added, incubated on ice for 10 min and centrifuged at 22,000xg for 10 min. The supernatant was removed and mixed with an equal volume of 100% ethanol by gentle inversion of the tubes. The sample incubated at room temperature for 10 min and was centrifuged at 22,000xg for 10 min. The pellet was washed twice with 70% ethanol, air-dried and resuspended in nuclease-free water. The quality and quantity of the extracted DNA was evaluated by ultraviolet spectroscopy.

DNA repeat element genotyping. The presence of repeat DNA elements in the human serotonin transporter (5HTT) and the dopamine-D4-receptor exon III cytosolic loop were determined by PCR amplification across these regions of genomic DNA and evaluating the size of the resulting DNA products by agarose gel electrophoresis. The PCR amplification primers for the 5HHT assay were 5'-ATGCCAGCACCTAACCCCTAATGT -3' (forward) and 5'-GGACCGCAAGGTGGGCGGGA-3' (reverse) (Steiger, H. et al, 2007), and those for the DRD4 assay were 5'- GCGACTACGTGGTCTACTCG -3' (forward) and 5'- AGGACCCTCATGGCCTTG -3' (reverse) (Lichter, JB et al, 1993). A PCR reaction (25 µl) containing 50 ng purified genomic DNA from each subject, 0.5 µM each of forward and reverse primers, 400 µM deoxyribonucleotides, PCR buffer, 1X Q-solution and Taq polymerase (1.25 units, QIAGEN) was performed in a 7900 thermocycler (Applied Biosystems, Inc.). The PCR conditions including the thermocycling conditions used for both assays were identical. We tested different polymerases, reagents (such as the Q solution that comes with the Taq polymerase from QIAGEN), primer pairs, annealing temperatures (at least 4 different ones for each assay) and number of cycles (35, 40 and 45). We also tested Taq polymerase from Invitrogen Corp as well as from QIAGEN. There is no difference between them and they can be used interchangeably. The samples were subjected to 95°C/5 min, 35 cycles of 94°C/30 sec, 60°C/30 sec and 72°C/1 min, and 72°C/4 min. After amplification, the PCR products were separated on a 2% agarose gel that was subsequently stained with ethidium bromide and visualized under ultraviolet light. Included in each assay was water as a negative control. Between 15-20% of the test samples were re-analyzed for data verification.

**Supplementary References and Notes**

Lichter JB, Barr CL, Kennedy JL, Van Tol HHM, Kidd KK et al. (1993) A hypervariable segment in the human dopamine receptor D4 (DRD4) gene. Human Molecular Genetics, 2(6): 767-773.

Steiger H, Richardson J, Joober R, Gauvin L, Israel M, et al. (2007) The 5HTTLPR polymorphism, prior maltreatment and dramaticerratic personality manifestations in women with bulimic syndromes. Journal of Psychiatry Neuroscience 32(5), 354-62.

Guo SW, Thompson EA (1992) **Performing the Exact Test of Hardy-Weinberg Proportion for Multiple Alleles.** Biometrics 48, 361-372.

The authors are grateful to Pastor Couceyro at ACGT Inc. for helpful comments and to Nick Bowman, Brandy Lipton and Agnieszka Tymula for excellent research assistance. Kuhnen acknowledges generous financial support for this work from the Zell Center for Risk Research at the Kellogg School of Management. This work is also supported by NSF BCS-0720312 and NSF BCS-0722326 grants to J.Y.C.
