# Supplementary material for: Genetic Determinants of Financial Risk Taking
Source: PLoS One. 2009 Feb 11;4(2):e4362. doi: 10.1371/journal.pone.0004362 (PMC2634960; doi:10.1371/journal.pone.0004362)
Supplement: Table S1 — Allele and genotype frequencies for 5HTTLPR polymorphism (0.03 MB DOC) [file pone.0004362.s002.doc]

**Supplementary Table 1.** Allele and genotype frequencies for 5HTTLPR polymorphism

| 5HTTLPR | n | % |
| --- | --- | --- |
| Allele |  |  |
| S | 52 | 40 |
| L | 78 | 60 |
| Total | 130 | 100 |
| Genotype |  |  |
| *s/s* | 21 | 32.3 |
| *s/l* | 10 | 15.4 |
| *l/l* | 34 | 52.3 |
| Total | 65 | 100 |
|  |  |  |
