# Supplementary material for: Genetic Determinants of Financial Risk Taking
Source: PLoS One. 2009 Feb 11;4(2):e4362. doi: 10.1371/journal.pone.0004362 (PMC2634960; doi:10.1371/journal.pone.0004362)
Supplement: Table S2 — Allele and genotype frequencies for DRD4 polymorphism (0.05 MB DOC) [file pone.0004362.s003.doc]

**Supplementary Table 2.** Allele and genotype frequencies for DRD4 polymorphism

| DRD4 | N | % |
| --- | --- | --- |
| Allele |  |  |
| 2 | 11 | 8.46 |
| 3 | 1 | 0.77 |
| 4 | 92 | 70.77 |
| 5 | 1 | 0.77 |
| 6 | 2 | 1.54 |
| 7 | 19 | 14.62 |
| 8 | 3 | 2.31 |
| 9 | 1 | 0.77 |
| Total | 130 | 100 |
| Genotype |  |  |
| 2/4 | 9 | 13.8 |
| 2/6 | 1 | 1.54 |
| 2/7 | 1 | 1.54 |
| 3/4 | 1 | 1.54 |
| 4/4 | 34 | 52.3 |
| 4/5 | 1 | 1.54 |
| 4/6 | 1 | 1.54 |
| 4/7 | 9 | 13.8 |
| 4/8 | 2 | 3.08 |
| 4/9 | 1 | 1.54 |
| 7/7 | 4 | 6.15 |
| 7/8 | 1 | 1.54 |
| 7- | 50 | 76.9 |
| 7+ | 15 | 23.1 |
| Total | 65 | 100 |
|  |  |  |
